# Loss of TDP-43 function contributes to genomic instability in amyotrophic lateral sclerosis

**DOI:** 10.3389/fnins.2023.1251228

**Published:** 2023-10-02

**Authors:** Minggang Fang, Sara K. Deibler, Alissa L. Nana, Sarat C. Vatsavayai, Shahid Banday, You Zhou, Sandra Almeida, Alexandra Weiss, Robert H. Brown, William W. Seeley, Fen-Biao Gao, Michael R. Green

**Affiliations:** ^1^Department of Molecular, Cell and Cancer Biology, University of Massachusetts Chan Medical School, Worcester, MA, United States; ^2^Department of Neurology, Memory and Aging Center, University of California, San Francisco, San Francisco, CA, United States; ^3^Department of Neurology, University of Massachusetts Chan Medical School, Worcester, MA, United States; ^4^RNA Therapeutics Institute, University of Massachusetts Chan Medical School, Worcester, MA, United States

**Keywords:** amyotrophic lateral sclerosis, frontotemporal dementia, TDP-43, DNA repair, genomic instability, homologous recombination

## Abstract

A common pathological hallmark of amyotrophic lateral sclerosis (ALS) and frontotemporal dementia (FTD) is the cytoplasmic mislocalization and aggregation of the DNA/RNA-binding protein TDP-43, but how loss of nuclear TDP-43 function contributes to ALS and FTD pathogenesis remains largely unknown. Here, using large-scale RNAi screening, we identify *TARDBP*, which encodes TDP-43, as a gene whose loss-of-function results in elevated DNA mutation rate and genomic instability. Consistent with this finding, we observe increased DNA damage in induced pluripotent stem cells (iPSCs) and iPSC-derived post-mitotic neurons generated from ALS patients harboring *TARDBP* mutations. We find that the increase in DNA damage in ALS iPSC-derived neurons is due to defects in two major pathways for DNA double-strand break repair: non-homologous end joining and homologous recombination. Cells with defects in DNA repair are sensitive to DNA damaging agents and, accordingly, we find that ALS iPSC-derived neurons show a marked reduction in survival following treatment with a DNA damaging agent. Importantly, we find that increased DNA damage is also observed in neurons with nuclear TDP-43 depletion from ALS/FTD patient brain tissues. Collectively, our results demonstrate that ALS neurons with loss of nuclear TDP-43 function have elevated levels of DNA damage and contribute to the idea that genomic instability is a defining pathological feature of ALS/FTD patients with TDP-43 pathology.

## 1. Introduction

Amyotrophic lateral sclerosis (ALS) is one of the major adult-onset neurodegenerative disorders, with a prevalence in the United States of 5 in 100,000 people (Mehta et al., 2022). The disease is characterized by the degeneration of motor neurons of the motor cortex, brainstem, and spinal cord, resulting in progressive muscle weakness, paralysis and eventual respiratory failure (Masrori and Van Damme, 2020). Approximately 10–15% of patients with ALS also develop frontotemporal dementia (FTD), marked by degeneration in the frontal, insular, and temporal cortex, resulting in a range of cognitive, behavioral and language deficits (Yang et al., 2020). The etiology of ALS is complex, with a combination of both genetic and environmental factors playing a role in the neurodegenerative process (Mejzini et al., 2019). Most cases of ALS are sporadic, whereas approximately 5–10% are caused by genetic mutations, most commonly a hexanucleotide repeat expansion in the *C9ORF72* gene or mutation in *SOD1*, *FUS* or *TARDBP* (Alsultan et al., 2016). In total, more than 50 genes associated with ALS risk and pathogenesis have been identified, and are involved in diverse biological functions (Mejzini et al., 2019). The large number of genes and cellular processes implicated in ALS has led to the notion that the pathophysiology of the disease is multifactorial, with several mechanisms contributing to neurodegeneration.

A major determinant of ALS pathogenesis is the *TARDBP* gene, which encodes transactive response DNA-binding protein 43 (TDP-43), a ubiquitously expressed DNA/RNA-binding protein that predominantly resides in the nucleus, but is capable of shuttling to the cytoplasm (Suk and Rousseaux, 2020). In the nucleus, TDP-43 plays diverse roles in gene regulation and RNA processing, with reported roles in transcription, RNA stability, microRNA biogenesis, and pre-mRNA splicing (Lagier-Tourenne et al., 2010; Liao et al., 2022). TDP-43 is a key component of RNA transport granules in neurons (Alami et al., 2014; Liu-Yesucevitz et al., 2014), and plays an important role in neuronal plasticity by regulating local protein synthesis in dendrites (Diaper et al., 2013). Mutations in *TARDBP* are rare in ALS and have been found in 1–5% of sporadic and familial ALS cases (Mackenzie et al., 2010b; Ravanidis et al., 2018). However, TDP-43 is a major component of cytoplasmic ubiquitin-positive protein inclusions that are the pathological hallmark of the disease and are found in ~97% of ALS patients regardless of genetic etiology (Neumann et al., 2006; Ling et al., 2013). The formation of TDP-43-positive cytoplasmic aggregates is almost always associated with clearing of TDP-43 from the nucleus and the presumptive loss of nuclear TDP-43 function (Neumann et al., 2006). Similar inclusions are seen in ~50% of patients with FTD (Neumann et al., 2006). A thorough understanding of the nuclear functions of TDP-43 is crucial to determining whether loss of these functions may play a role in disease pathogenesis.

Similar to other age-related neurodegenerative disorders, ALS/FTD is associated with an impaired DNA damage response and resultant increase in genomic instability, which is thought to contribute to motor neuron degeneration and disease pathogenesis (Konopka and Atkin, 2022). Indeed, many ALS-associated genes have roles in the DNA damage response, and defects in these genes lead to deficiencies in DNA repair (Sun et al., 2020). For example, mutations in *C9ORF72* are associated with increased DNA double-strand breaks and defects in ATM-mediated repair in cells and spinal cord tissues from *C9ORF72*-ALS patients (Lopez-Gonzalez et al., 2016; Farg et al., 2017; Walker et al., 2017) and induce neurodegeneration in mice (Walker et al., 2017). Moreover, ALS-associated mutations in the *FUS* gene lead to defects in DNA repair (Wang et al., 2013) and increase DNA damage in mice (Qiu et al., 2014). Several studies have implicated a role for TDP-43 in maintaining genomic stability through diverse mechanisms including promoting non-homologous end-joining DNA repair (Mitra et al., 2019; Konopka et al., 2020) and preventing accumulation of genotoxic R-loops (Hill et al., 2016; Giannini et al., 2020; Wood et al., 2020). Defective DNA repair and genomic instability represent a potential unifying etiology that underlies the diverse pathophysiological mechanisms of ALS/FTD (Sun et al., 2020).

Here, using an unbiased, large-scale loss-of-function screening approach, we identify *TARDBP* as a gene required for genomic stability. We find that post-mitotic neurons derived from ALS patient induced pluripotent stem cells (iPSCs) harboring *TARDBP* mutations are defective in DNA repair and display increased DNA damage, which we also observe in neurons lacking nuclear TDP-43 in ALS/FTD patient brain tissues. We show that the increase in DNA damage in ALS iPSC-derived neurons is due to defects in two major pathways for DNA double-strand break repair. Our results provide support for the model that loss of nuclear TDP-43 function underlies genomic instability and molecular pathogenesis in ALS/FTD.

## 2. Materials and methods

### 2.1. Mutation rate screen and validation assays

The RNAi Consortium (TRC) lentiviral mouse shRNA library (Dharmacon/Horizon Discovery), divided into 24 pools (5,000 shRNAs per pool), was obtained through the UMass Chan Medical School RNAi Core Facility. The lentiviral pools were generated with titers of ~2–3 × 10^7^ cfu/mL, as previously described (Gazin et al., 2007). Because ethyl methanesulfonate (EMS) treatment alone will result in a small number of *Aprt^−/−^* cells, a pilot experiment was performed to determine the minimal number of *Aprt^−/−^* 3C4 cells required to form colonies in the presence of 2,6-diaminopurine. Toward this end, 1 × 10^4^, 1 × 10^5^, or 1 × 10^6^ 3C4 cells expressing a non-silencing (NS) negative control shRNA, or as a positive control an shRNA targeting the DNA mismatch repair gene *Mlh1*, whose inactivation is known to increase mutation rate (Hsieh and Yamane, 2008), were treated with EMS and then cultured in the presence of 2,6-diaminopurine for 10 days to select for *Aprt^−/−^* cells. In the presence of EMS, 1 × 10^4^ *Mlh1* knockdown 3C4 cells were sufficient to form colonies, whereas 3C4 cells expressing a control NS shRNA did not form any colonies (see Supplementary Figure S1A). Thus, for an shRNA to be represented in 1 × 10^4^ cells to form a colony, a minimum of 5 × 10^7^ cells would need to be transduced. For the primary screen, 5 × 10^7^ mouse embryonic stem cells (clone 3C4; provided by Jay Tischfield, Rutgers University), maintained in embryonic stem cell culture as previously (Cervantes et al., 2002), were transduced with each pool of the shRNA library at an MOI of 0.2 and selected with 1.5 μg/mL puromycin for 4 days. Cells were then treated with EMS (Sigma, 300 μg/mL) for 5 h, after which the cells were washed with media. Cells were plated on 100-mm dishes, incubated with media containing 2,6 di-aminopurine (Sigma, 5 μg/mL). After 10 days, DAP-resistant colonies were harvested for genomic DNA isolation, and shRNAs were identified by sequence analysis as previously described (Gazin et al., 2007).

For validation assays, individual knockdown cell lines were generated by stable transduction of mouse 3C4 cells (1 × 10^6^) or human A549 cells (1 × 10^5^, obtained from the ATCC) with a single shRNA (Supplementary Table S1) followed by puromycin selection and incubation with 5 μg/mL 2,6 di-aminopurine (for 3C4 cells) or 0.75 μg/mL 6-thioguanine (Sigma) (for A549 cells) for 10 days. Surviving colonies were fixed with 50% ethanol, stained with crystal violet, and counted. Mutation rate was calculated by the P0 method as previously described (Cervantes et al., 2002) and corrected for colony forming efficiency. Rates are presented per cell per generation.

### 2.2. qRT-PCR

Total RNA was isolated, and reverse transcription was performed as described (Gazin et al., 2007), followed by qRT-PCR using Power SYBR Green PCR Master Mix (Applied Biosystems) and *TDP-43* gene-specific primers (forward, 5′-ATTCAAAGGGGTTTGGCTTT-3′, and reverse, 5′-CAGTCACACCATCGTCCATC-3′). *GAPDH* was used as an internal reference gene for normalization.

### 2.3. Human ALS patient-derived iPSCs and neurons

Human control iPSCs [clones 2#20 (WT-1), 37#20 (WT-2) and 35#11 (WT-3)] (Almeida et al., 2012; Zhang et al., 2013; Freibaum et al., 2015) and ALS patient-derived iPSCs (clones HPS0292 ((M337V)-1), HPS0293 ((M337V)-2), HPS0290 ((Q343R)-1) and HPS0291 ((Q343R)-1)) (Egawa et al., 2012), provided by the RIKEN BRC through the National BioResource Project of the MEXT/AMED, Japan, were cultured in mTeSR1 media (STEMCELL Technologies) on matrigel-coated plates. To generate iPSC-derived neurons, iPSCs were differentiated by lentiviral gene transfer and genomic integration of doxycycline-inducible Neurogenin1/2 and rTA3 vectors as previously described (Busskamp et al., 2014). To confirm neuronal differentiation, neurons were fixed with 4% paraformaldehyde in PBS for 10 min, blocked with 10% normal goat serum (Vector Laboratories), and then stained with a TUJ1 (Covance) antibody for 1 h at room temperature. Cells were then rinsed several times with PBS, incubated with an Alexa 488-conjugated donkey anti-mouse secondary antibody (Molecular Probes) and DAPI (Molecular Probes) in the appropriate buffer for 1 h at room temperature. After several more rinses, cells were mounted with Vectashield (Vector Laboratories) and imaged with a Zeiss Axiovert microscope and 10X objective equipped with a Zeiss Axiocam digital camera.

### 2.4. Comet assays

Control or mutant TDP-43 ALS iPSC or iPSC-derived neurons at day 20 were harvested, counted for comet assays. Single-cell gel electrophoresis under alkaline conditions was performed using a Comet assay kit (Trevigen, 4250-050-K). Samples were stained with SYBR-green and observed using a Zeiss AXIO Imager Z2 microscope. Images were analyzed using ImageJ software.

### 2.5. NHEJ and HR reporter assays

The small-plasmid-based NHEJ assay was performed as previously described (Wilson et al., 1999). Briefly, iPSC-derived neurons (at day 4 of the differentiation process) were co-transfected with a linearized small ampicillin plasmid (pGL-4.14) and a circularized kanamycin plasmid (pDSRed-N1). Forty-eight hours post-transfection, plasmids were isolated and transformed into *E. coli* strain DH10B. The NHEJ frequency was determined by calculating the ratio of ampicillin-resistant colonies to kanamycin-resistant colonies, and the result was normalized to that observed in control neurons, which was set to 1. The chromosomal NHEJ assay was performed with HEK293/pPHW1 cells as previously described (Zhuang et al., 2009). The NHEJ frequency was calculated from the ratio of the number of XHATM-resistant colonies to the total number of cells seeded and normalized for transfection and plating efficiencies.

The small-plasmid-based HR assay was performed as previously described (Thyagarajan et al., 1996; Fang et al., 2013). Briefly, plasmids pSV2neoDL, pSV2neoDR, and pRSVed1884 were transfected into iPSC-derived neurons (at day 4 of the differentiation process) using an Amaxa Cell Line Optimization Nucleofector Kit (Lonza). Forty-eight hours post-transfection, plasmids were isolated, digested with DpnI, and transformed into *E. coli* strain DH10B. The HR frequency was determined by calculating the ratio of kanamycin-resistant colonies to ampicillin-resistant colonies, and the result was normalized to that observed in control neurons, which was set to 1. The chromosomal HR assay was performed with HCT116/HN5 cells as previously described (Mohindra et al., 2004; Wen et al., 2008). The HR frequency was calculated from the ratio of the number of G418-resistant colonies to the total number of cells seeded and normalized for transfection and plating efficiencies.

### 2.6. RAD51 foci formation

Immunocytochemical analysis was performed as previously described (Sorensen et al., 2005). Briefly, cells were treated in the presence or absence of 0.2 mM hydroxyurea (Sigma) for 24 h. Cells were stained with anti-RAD51 (Santa Cruz) and 4,6-diamidino-2-phenylindole (DAPI) and analyzed on a Zeiss AXIO Imager Z2 microscope. Nuclei with >5 foci were considered positive for hydroxyurea-induced RAD51 foci. The percentages of cells with positive RAD51 were determined in at least two separate experiments by counting at least 300 nuclei on each slide.

### 2.7. Survival assays with etoposide and 5-fluorouracil

Control or mutant TDP-43 ALS iPSCs (5 × 10^5^) were seeded in 60-mm dishes, and 24 h later incubated with a range of concentrations of etoposide (Sigma) or 5-fluorouracil (Sigma) for 14 days. Colonies were fixed, stained with crystal violet and counted. Control or mutant TDP-43 ALS iPSC-derived neurons (1 × 10^5^) at day 20 were seeded in one well of 6-well plates, 24 hours later were incubated with a series of concentration of etoside or 5-fluorouracil for 3 days. Neuron viability was measured with alamarBlue assay (Thermo Fisher Scientific). The results were normalized to 0 uM (DMSO solvent), which was set to 1.0.

### 2.8. Patient information and neuropathological assessment

Post-mortem human brain tissue samples were obtained from the UCSF Neurodegenerative Disease Brain Bank. Consent for brain donation was obtained from all subjects or their surrogates in accordance with the Declaration of Helsinki, and the research was approved by the University of California, San Francisco Committee on Human Research. Clinical diagnoses of bvFTD were made according to prevailing international consensus criteria at the time of assessment (Neary et al., 1998; Rascovsky et al., 2011). Brains were cut fresh into 8–10 mm thick coronal slabs and immersion fixed on a platform in 10% neutral buffered formalin (Fisher Scientific, United States). Neuropathological diagnoses were made following consensus diagnostic criteria (Mackenzie et al., 2010a, 2011; Montine et al., 2012) using previously described immunohistochemical and histological methods (Tartaglia et al., 2010). Four patients with FTLD-TDP were selected per brain region (2 sporadic and 2 carrying the *C9ORF72* expansion) based on clinical and neuropathological diagnoses (Supplementary Table S2).

### 2.9. Immunofluorescence staining

Eight-micrometer thick sections of precentral gyrus were cut from formalin fixed paraffin-embedded tissue blocks taken for neuropathological diagnosis (Vatsavayai et al., 2016). Blocks of the frontoinsula were dissected from fixed slabs, cryoprotected, and sectioned at 50 μm as previously described (Kim et al., 2012; Nana et al., 2019). DNA damage was assessed using an antibody against phosphorylated-Serine139 to Histone H2AX (H2AX, mouse, 1:5 k, Millipore Sigma 05-636). An antibody against TDP-43 (rabbit, 1:4,000, Proteintech 10782-2-AP) was used to identify neurons lacking normal nuclear TDP-43 and bearing a TDP-43 cytoplasmic inclusion, and layer 5 neurons were identified with MAP2 (chicken, 1:3,000, EnCor Biotechnology Inc. CPCA-MAP2) using previously described immunofluorescence methods (Nana et al., 2019). All sections were counterstained with NeuroTrace 435/455 Blue Fluorescent Nissl Stain (Invitrogen).

### 2.10. Image capture and analysis

**Figure 1:**
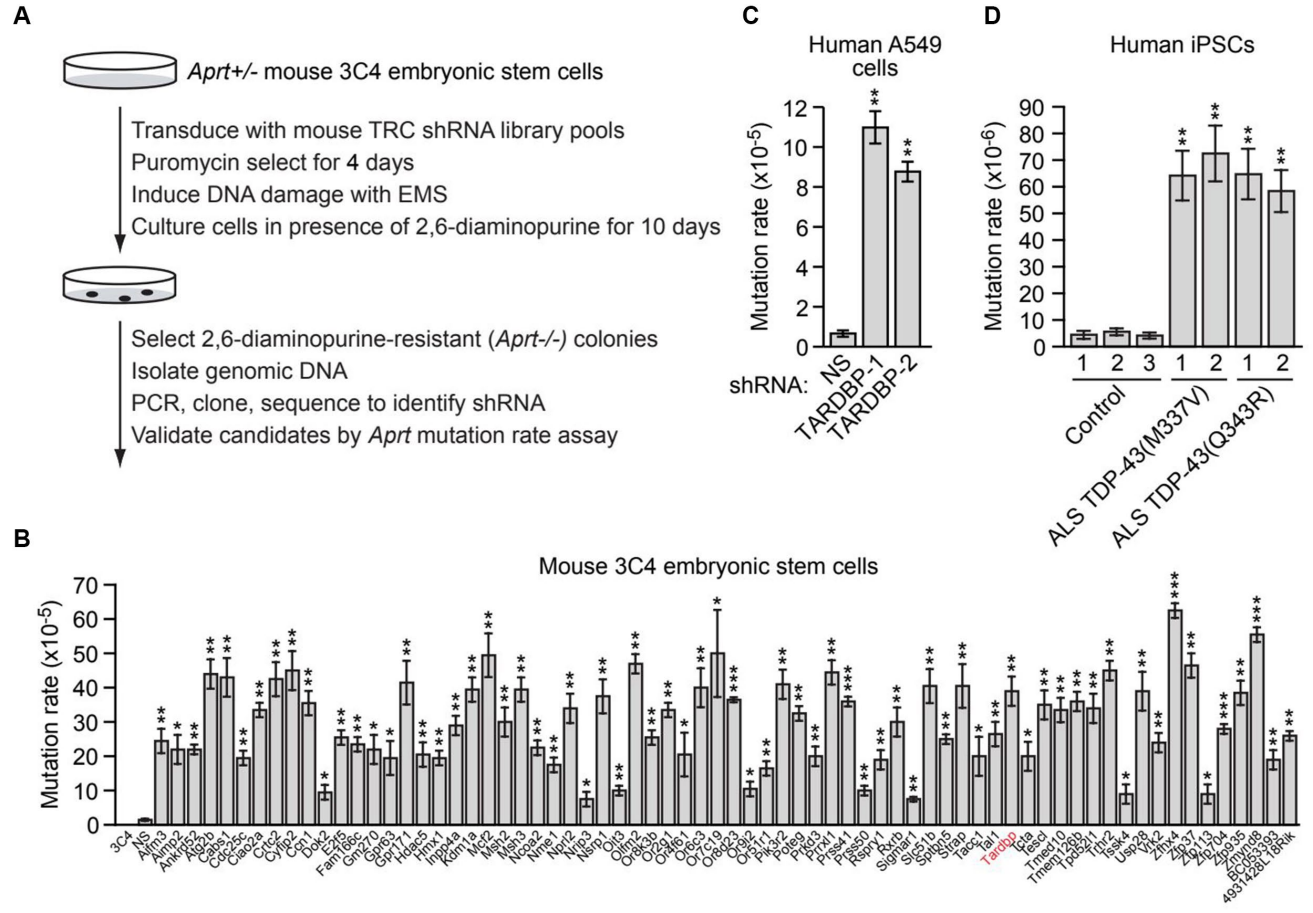
A large-scale RNAi screen identifies genes required for genomic stability, including *TARDBP*. **(A)** Schematic summary of the large-scale RNAi screening strategy. **(B)** *Aprt* mutation rate in mouse 3C4 embryonic stem cells expressing an shRNA targeting one of 70 genes identified in the initial RNAi screen. Shown as controls are parental 3C4 cells, and 3C4 cells expressing a non-silencing (NS) shRNA. Not shown are the results with an *Aprt* shRNA, which, as expected, was isolated in the primary screen. **(C)** Spontaneous *HPRT* mutation frequency in A549 cells expressing a NS shRNA or one of two unrelated *TARDBP* shRNAs, *TARDBP-1* and *TARDBP-2*. **(D)** Spontaneous *HPRT* mutation frequency in iPSC lines generated from two familial ALS patients harboring a TDP-43(M337V) or TDP-43(Q343R) mutation, or in three control iPSC lines. Data are represented as mean ± SD; Statistical analysis was done with Students’ t test, *p < 0.05, **p < 0.01, ***p < 0.001.

**Figure 2:**
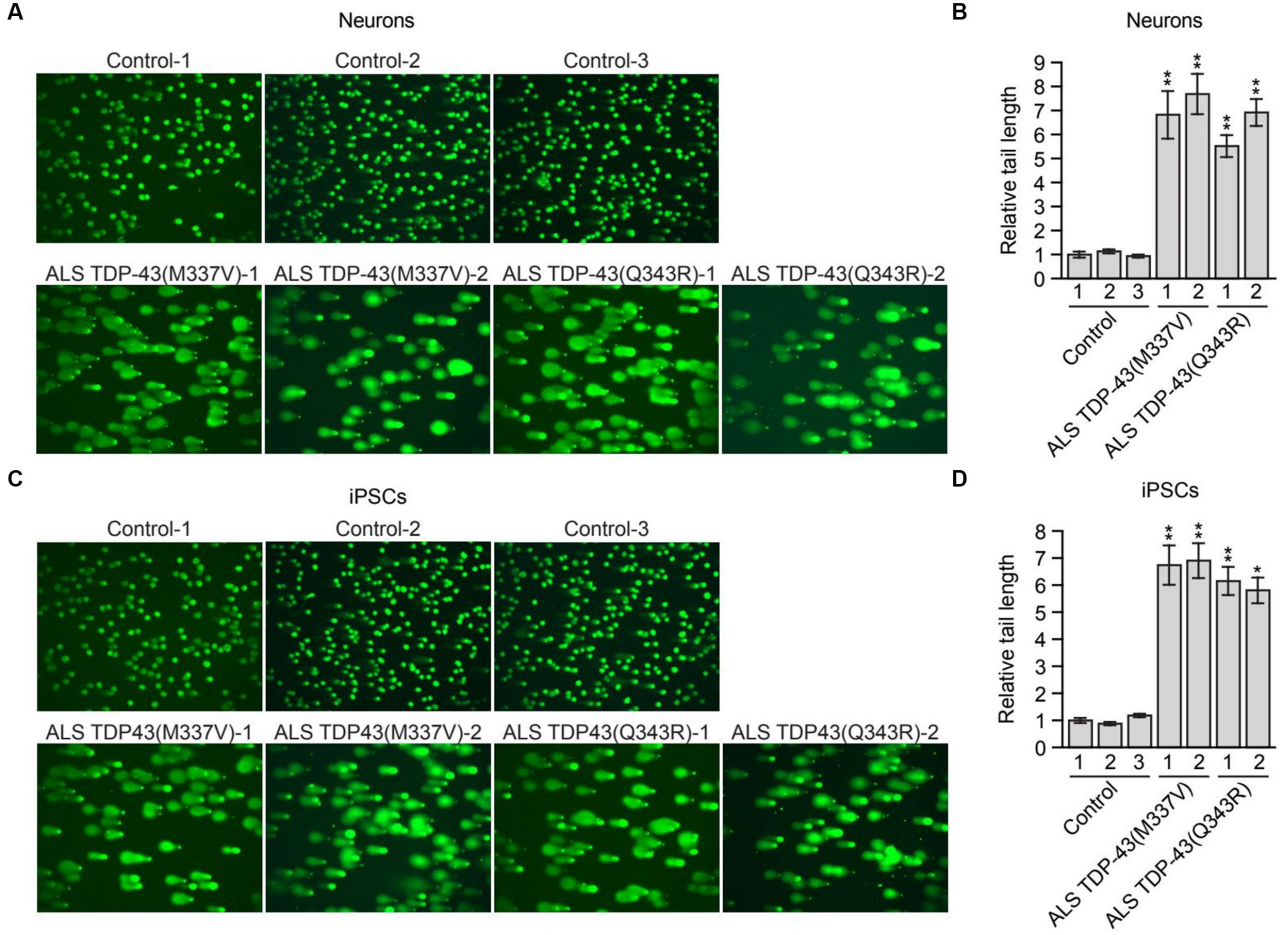
Mutant TDP-43 ALS iPSC-derived neurons have elevated levels of DNA damage. **(A)** Comet assay in control, TDP-43(M337V) or TDP-43(Q343R) ALS iPSC-derived neurons. Representative images are shown. **(B)** Quantification of the comet assays shown in **(A)**. **(C)** Comet assay in control, TDP-43(M337V) and TDP-43(Q343R) ALS iPSCs. Representative images are shown. **(D)** Quantification of the comet assays shown in **(C)**. Data are represented as mean ± SD; Statistical analysis was done with Students’ t test, *p < 0.05, **p < 0.01.

For the precentral gyrus, non-overlapping Z-stack images at x60 magnification were captured in layer 5 based on Nissl and MAP2 channels and blind to TDP-43 and γH2AX status on a Zeiss LSM 880 confocal laser scanning microscope. For the FI, to ensure image capture of at least one TDP-inclusion bearing neuron per image, 20 TDP-inclusion-bearing MAP2-positive neurons from layer 5 were marked at random at x10 magnification using only the Nissl, MAP2, and TDP-43 channels, then z- stacks were captured at each marker location at x40 magnification from all four channels. Neurons from both regions were counted manually using Zeiss Zen software. For each field of view, investigators first marked all neurons using the MAP2 channel only. Next, each neuron was classified as TDP-43 inclusion-bearing or normal, blinded to γH2AX status. The TDP-43 channel and markers were then hidden, and each neuron was assessed for the presence of γH2AX foci. γH2AX staining showed two patterns of immunoreactivity: (1) 1–3 large punctate γH2AX positive foci in the nucleus (Figure 1A) or (2) a diffuse pan-nuclear γH2AX staining. Given that in brain tissue focal but not pan-nuclear γH2AX staining is associated with DNA DSBs (Shanbhag et al., 2019), we only counted neurons with nuclear foci. A total of 285 neurons from precentral gyrus and 979 neurons from frontoinsular cortex were counted.

Statistical analysis was performed using R version 4.0.5 in RStudio 1.1.1093 using logistic regression (binomial glm) in the lme4 package (http://www.r-project.org). The presence or absence of γH2AX foci was used as the outcome, and TDP-43 inclusion status as the primary categorical predictor, with *C9ORF72* status and subject as covariates. A separate model was run for each region. A *p* < 0.05 (two-tailed) was considered statistically significant.

## 3. Results

### 3.1. A large-scale RNAi screen identifies genes required for genomic stability, including TARDBP

To identify new genes required for genomic stability, we performed a large-scale loss-of-function RNAi screen for genes that, when knocked down, result in an increased mutation rate. For the screen readout, we used a well-established genotoxic assay based on loss of heterozygosity of the adenine phosphoribosyl transferase (*Aprt*) gene, which confers resistance to the drug 2,6-diaminopurine. The screen was performed in mouse 3C4 embryonic stem cells, which are heterozygous for the *Aprt* gene (*Aprt^+/−^*) (Cervantes et al., 2002). Previous studies have shown that the spontaneous mutation rate at *Aprt* in these cells is relatively low (8.3 × 10^−8^) (Cervantes et al., 2002), therefore the primary screen was performed in the presence of the chemical mutagen ethyl methanesulfonate (EMS) to increase the mutation rate and reduce the number of cells required for large-scale screening. For the primary screen, a genome-wide mouse short hairpin RNA (shRNA) library was divided into 24 pools (5,000 shRNAs per pool), which were then packaged into lentivirus particles to stably transduce 5 × 10^7^ 3C4 cells so that on average, each shRNA was represented in a sufficient number of cells that could enable colony formation (Supplementary Figure S1A). Cells were treated with EMS and then cultured in the presence of 2,6-diaminopurine for 10 days to select for *Aprt^−/−^* cells, which were then harvested and subjected to sequence analysis to identify the shRNAs (Figure 1A). Individual positive candidate shRNAs were validated in a second *Aprt* mutation rate assay performed in the absence of EMS, in which the number of 2,3-diaminopurine-resistant colonies were counted and used to calculate the spontaneous mutation rate. Using this approach, we identified 70 genes whose knockdown significantly increased the spontaneous mutation rate ≥2-fold compared to a control non-silencing (NS) shRNA (Figure 1B). The 70 genes encode proteins that are involved in a variety of biological processes, including apoptosis and autophagy, cell cycle regulation, signal transduction, and transcription (Supplementary Table S3). Notably, the screen identified the mismatch repair genes *Msh2* and *Msh*3, whose inactivation is known to increase mutation rate (Hsieh and Yamane, 2008). The screen also identified several other factors known to play a role in genomic stability, such as *Aimp2* (Han et al., 2008), *Atg2b* (Kang et al., 2009), *Cdc25c* (Thanasoula et al., 2012), *Crtc2* (Fang et al., 2015), *Kdm1a* (Mosammaparast et al., 2013), *Nme1* (Kaetzel et al., 2015), *Nprl2* (Ma et al., 2017), *Tardbp* (Hill et al., 2016; Mitra et al., 2019; Giannini et al., 2020; Konopka et al., 2020), *Usp28* (Zhang et al., 2006), and *Zmynd8* (Gong et al., 2015), demonstrating the validity of our experimental approach. Validation of a representative subset of candidates using a second, unrelated shRNA confirmed that target gene knockdown increased spontaneous *Aprt* mutation rate (Supplementary Figure S1B).

We elected to focus our further investigations on *TARDBP*. To confirm the elevated mutation rate in *TARDBP*-knockdown cells, we performed a second mutation rate assay based on hypoxanthine-guanine phosphoribosyltransferase (HPRT) deficiency. In this assay, human lung adenocarcinoma A549 cells harboring mutations in the essential *HPRT* gene are detected by positive selection using 6-thioguanine (6-TG). We found that knockdown of *TARDBP* using one of two unrelated shRNAs (Supplementary Figure S1C) significantly increased the spontaneous *HPRT* mutation rate (Figure 1C).

Because of the importance of TDP-43 in ALS/FTD pathophysiology, we sought to confirm and extend our results in ALS patient cells. We therefore measured the spontaneous mutation rate in four induced pluripotent stem cell (iPSC) lines generated from two familial ALS patients harboring a heterozygous TDP-43(M337V) or TDP-43(Q343R) mutation (Egawa et al., 2012), and in three control iPSC lines (Zhang et al., 2013). Figure 1D shows that the *HPRT* mutation rate in iPSCs containing TDP-43(M337V) or TDP-43(Q343R) (hereafter called mutant TDP-43 ALS iPSCs) was substantially higher than that observed in control iPSCs, indicating increased genomic instability in ALS patient cells harboring TDP-43 mutations.

### 3.2. Mutant TDP-43 ALS iPSC-derived neurons have elevated levels of DNA damage

Previous studies have shown that loss of nuclear TDP-43 function results in reduced DNA repair and elevated levels of DNA damage (Mitra et al., 2019; Konopka et al., 2020). These prior studies were performed in TDP-43-depleted neuronal cell lines, normal iPSCs and iPSC-derived neural progenitor cells (Mitra et al., 2019) or in neuron-like cells transfected with plasmids expressing TDP-43 mutants (Konopka et al., 2020). To determine whether increased DNA damage is also observed in ALS patient neurons harboring *TARDBP* mutations, we performed a comet assay, a sensitive method to evaluate DNA lesions at a single-cell level (Olive and Banath, 2006). For these experiments, we differentiated control and mutant TDP-43 ALS iPSCs into neurons as previously described (Busskamp et al., 2014), and confirmed neuronal differentiation by staining for the neuronal marker TUJ1 (Supplementary Figure S2A). As expected, very few comet tails were observed in neurons differentiated from multiple control iPSC lines, indicating a very low level of endogenous DNA lesions (Figure 2A). By contrast, mutant TDP-43 ALS neurons differentiated from multiple patient iPSC lines displayed a significant increase in comet tail length (Figures 2A,B). To examine if the increased DNA damage upon TDP43 mutation is specific to neuron or common to other cell types, TDP-43 mutant iPSC cells were also examined. Similarly, mutant TDP-43 ALS iPSCs also showed increased comet tail length compared to control iPSCs (Figures 2C,D). As expected, an increase in comet tail length was also observed in control neurons following shRNA-mediated knockdown of *TARDBP* (Supplementary Figure S2B).

**Figure 3:**
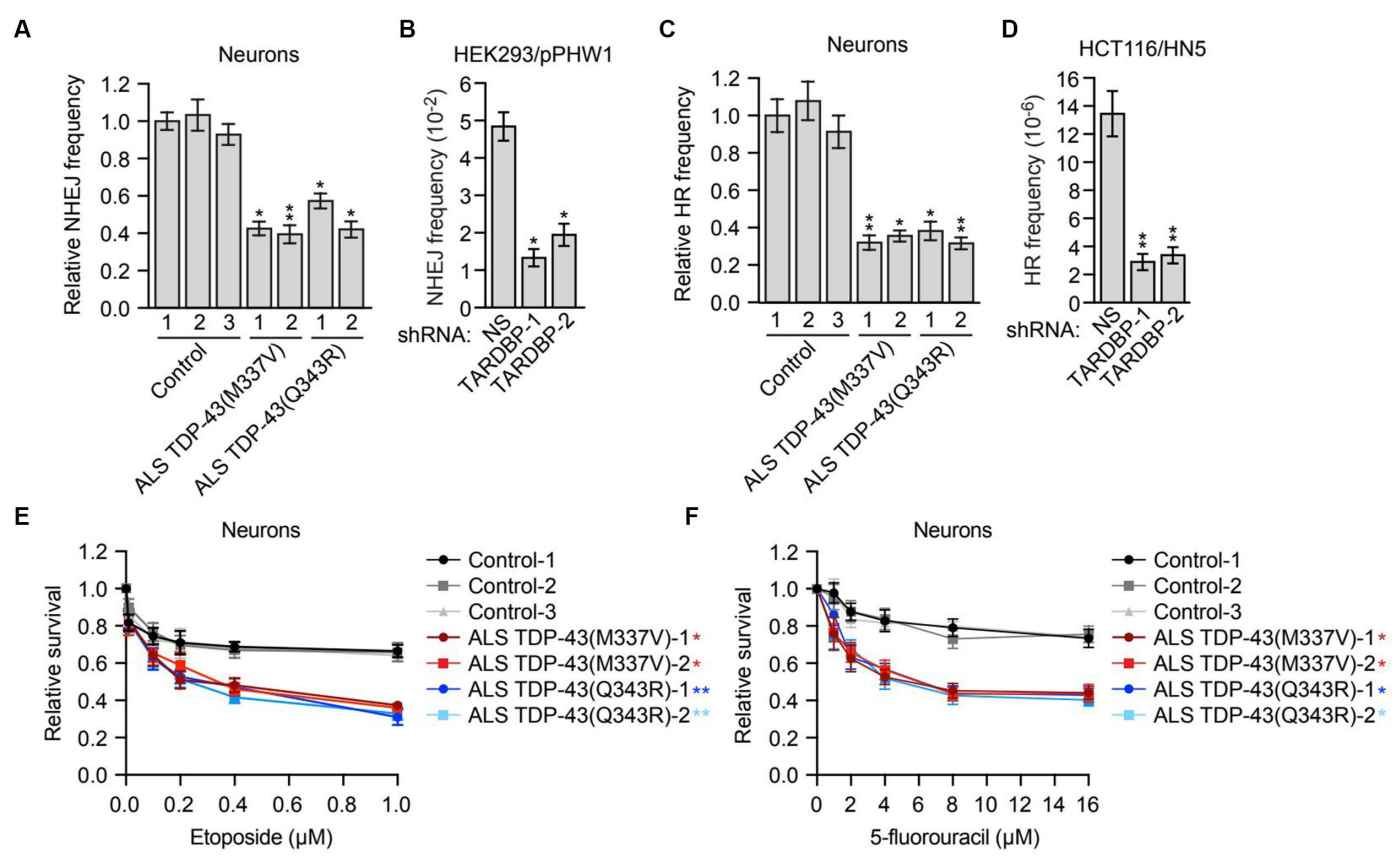
Mutant TDP-43 ALS iPSC-derived neurons are deficient in NHEJ and HR DNA repair pathways and are sensitive to DNA damaging agents. **(A)** Plasmid-based NHEJ assay in ALS iPSC-derived neurons containing TDP-43(M337V) or TDP-43(Q343R). The results were normalized to that obtained in control neurons, which was set to 1. **(B)** Chromosomal-based NHEJ assay in HEK293/pPHW1 cells expressing a NS or *TARDBP* shRNA. **(C)** Plasmid-based HR assay in control, TDP-43(M337V) or TDP-43(Q343R) ALS iPSC-derived neurons. **(D)** Chromosomal-based HR assay in HCT116/HN5 cells expressing a NS or *TARDBP* shRNA. **(E,F)** Survival curves for control, TDP-43(M337V) or TDP-43(Q343R) ALS iPSC-derived neurons treated with increasing doses of etoposide **(E)** or 5-fluorouracil **(F)**. The results were normalized to those obtained in the absence of drug, which was set to 1. Data are represented as mean ± SD; Statistical analysis was done with Students’ t test, *p < 0.05, **p < 0.01. In **(E,F)**, *p* value comparisons are shown for treatment with 1 μM etoposide and 16 μM 5-fluorouracil, respectively.

### 3.3. Mutant TDP-43 ALS neurons are deficient in NHEJ and HR DNA repair pathways and are sensitive to DNA damaging agents

In mammalian cells, there are two major pathways for DNA double-strand break repair: non-homologous end joining (NHEJ) and homologous recombination (HR) (Chapman et al., 2012). As mentioned above, previous studies have shown that depletion of TDP-43 results in reduced NHEJ repair (Mitra et al., 2019). To investigate whether, as expected, NHEJ is impaired in mutant TDP-43 ALS neurons, we first used a well-established assay that is based upon NHEJ-directed repair of a plasmid-borne ampicillin-resistance reporter gene (Wilson et al., 1999). Briefly, control or mutant TDP-43 ALS neurons were co-transfected with a linearized plasmid encoding an ampicillin-resistance gene and a circularized plasmid encoding a kanamycin-resistance gene. Generation of a functional ampicillin-resistance gene is dependent on NHEJ. Following isolation of the plasmids and transformation into bacteria, NHEJ frequency was calculated as the ratio of ampicillin-resistant (indicative of repair) to kanamycin-resistant (indicative of total plasmid recovery) colonies. We found that NHEJ activity in mutant TDP-43 ALS neurons was ~2-fold lower than that in control neurons (Figure 3A). We also performed a chromosomal-based NHEJ assay in which *TARDBP* was knocked down in HEK293/pPHW1 cells, which contain a non-functional integrated *GPT* reporter gene that can be repaired by NHEJ (Zhuang et al., 2009). We found that shRNA-mediated knockdown of *TARDBP* in HEK293/pPHW1 cells (Supplementary Figure S3A) resulted in a 2.5–4-fold decrease in NHEJ activity (Figure 3B).

**Figure 4:**
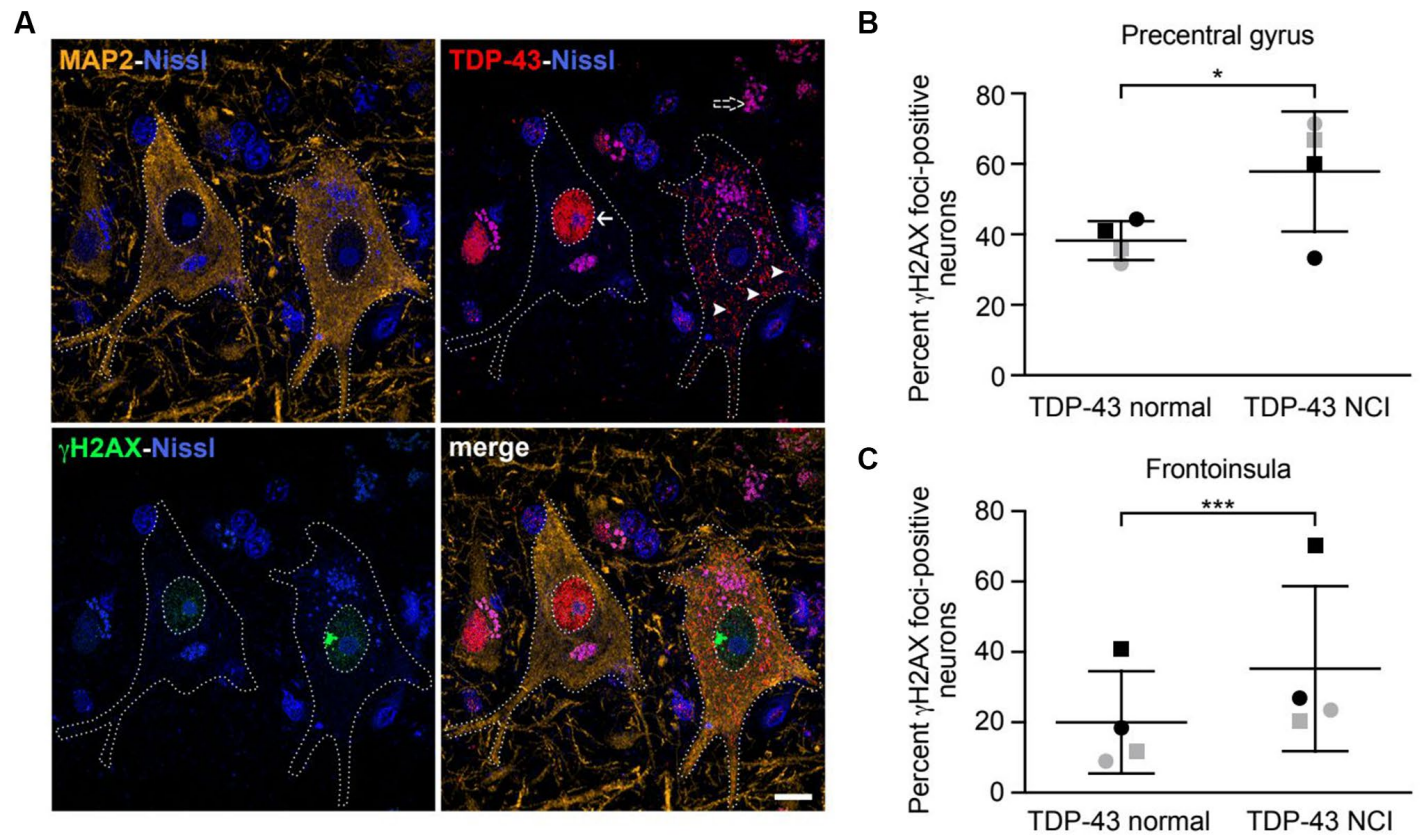
Neurons with nuclear TDP-43 depletion are associated with increased DNA damage in ALS/FTD patient brain tissues. **(A)** Confocal images showing two representative upper motor neurons from the precentral gyrus with either normal nuclear TDP-43 (arrow) or loss of nuclear TDP-43 and accompanying cytoplasmic inclusions (arrowheads) with a large γH2AX-positive focus in the nucleus, consistent with DNA double-strand breakage. Dotted lines represent cell and nuclear boundaries derived from the MAP2 immunostaining. All sections were counterstained with fluorescent Nissl stain (blue). Purple granular staining (dashed arrow) is lipofuscin autofluorescence background seen across channels. Images are maximum intensity projections of a confocal z-stack. Scale bar equals 10 μm. **(B,C)** Percentage of neurons showing γH2AX foci in cells with normal nuclear TDP-43 or TDP-43 neuronal cytoplasmic inclusions (NCIs) in the precentral gyrus **(B)** and frontoinsula **(C)** of patients with either *C9ORF72*-associated (gray) or sporadic (black) FTD-ALS. Data are represented as mean ± SD; Statistical analysis was performed using R version 4.0.5 in RStudio 1.1.1093, *p < 0.05, ***p < 0.001.

Although in post-mitotic neurons the prevalent DNA double-strand break repair pathway is NHEJ (Merlo et al., 2016), several reports have shown that HR also occurs in neurons (Cox et al., 2015; Welty et al., 2018; Yu et al., 2018). We therefore tested whether HR is also affected in mutant TDP-43 ALS neurons. First we used a plasmid-based assay similar to that described above in which two plasmids carrying non-overlapping deletions within the coding region of the kanamycin-resistance gene are co-transfected into cells, and generation of a functional kanamycin gene is dependent on inter-plasmid HR (Thyagarajan et al., 1996). We found that the HR frequency was 2–3-fold lower in mutant TDP-43 ALS neurons compared to control neurons (Figure 3C). To confirm this result, we also carried out a chromosomal-based HR assay. We knocked down *TARDBP* in HCT116/HN5 cells (Supplementary Figure S3B), a reporter cell line that contains two chromosomally-integrated non-functional portions of a neomycin-resistance gene (Mohindra et al., 2004; Wen et al., 2008). HR can generate an intact neomycin-resistance gene, enabling colony formation in the presence of neomycin. We found that shRNA-mediated knockdown of *TARDBP* resulted in a 4–5-fold decrease in HR efficiency (Figure 3D).

To further investigate the possibility that loss of TDP-43 impairs HR activity, we analyzed RAD51 foci formation, a characteristic marker of HR-directed DNA repair (Moynahan and Jasin, 2010). Human A549 cells stably expressing a *TARDBP* shRNA were subjected to DNA replication stress by the addition of hydroxyurea, a DNA replication inhibitor, and 24 h later, RAD51 foci were detected by immunofluorescence. We found that following *TARDBP* knockdown, there was a substantial decrease in the percentage of RAD51 foci-positive cells (Supplementary Figure S3C).

Cells that are defective for DNA repair are sensitive to DNA damaging agents (Bouwman and Jonkers, 2012). We therefore measured the relative sensitivity of control and mutant TDP-43 ALS neurons to DNA damage. Using increasing doses of the DNA damaging agent etoposide (Figure 3E) or 5-fluorouracil (Figure 3F), we generated survival curves for control and mutant TDP-43 ALS neurons and found that compared to control neurons, mutant TDP-43 ALS neurons displayed a reduction in survival following treatment with either DNA damaging agent. Similar results were obtained in iPSCs (Supplementary Figure S4). Collectively, these results indicate that mutant TDP-43 ALS neurons in culture are deficient in both NHEJ and HR repair pathways, leading to elevated levels of DNA damage.

### 3.4. Neurons with nuclear TDP-43 depletion show increased DNA damage in ALS/FTD patient brain tissues

A previous report found increased DNA double-strand breaks in spinal cord tissues from ALS patients with TDP-43 pathology (i.e., formation of TDP-43-positive cytoplasmic inclusions and nuclear TDP-43 depletion) compared to matched controls (Mitra et al., 2019). However, whether increased DNA damage is observed in the same neurons that have TDP-43 pathology had not been carefully examined. We therefore obtained brain tissues from patients with either *C9ORF72* mutations or sporadic FTD-ALS, both of which were accompanied by typical TDP-43 pathology. ALS is characterized by loss of upper motor neurons of the motor cortex, the majority of which are located in layer 5 (Ragagnin et al., 2019). FTD is associated with degeneration targeting a similar population of large, layer 5 projection neurons within the frontoinsular and anterior cingulate cortices (Nana et al., 2019). We therefore investigated layer 5 neurons within the precentral gyrus (targeted in ALS) and frontoinsular cortex (targeted in FTD), in patients with a blended FTD-ALS presentation. We combined immunofluorescence staining for γH2AX, a widely used marker for DNA double-strand breaks (Sedelnikova and Bonner, 2006), and TDP-43 (Figure 4A). Cells were also co-stained with the neuronal marker MAP2. In precentral gyrus, γH2AX foci were observed in 62% of TDP-43 inclusion-positive neurons (*n* = 26) compared to 38% of neurons with normal nuclear TDP-43 (*n* = 259) (Figure 4B). Similarly, in the frontoinsula, γH2AX foci were observed in 35% of TDP-43 inclusion-positive neurons (*n* = 139) compared to 21% in neurons without TDP-43 pathology (*n* = 782) (Figure 4C). In both regions, we found a significant increase in γH2AX foci in neurons with depleted nuclear TDP-43 and cytoplasmic aggregation (precentral gyrus *z* = 2.275, *p* = 0.0229; frontoinsula *z* = 3.991, *p* < 0.001). Collectively, these results indicate that neurons with loss of nuclear TDP-43 function display increased DNA double-strand breaks in patient brain tissues.

## 4. Discussion

In this study, we identified *TARDBP* in an unbiased large-scale RNAi screen for genes involved in maintaining genomic stability, as assessed by increased mutation frequency. In addition to *TARDBP*, the screen identified several other genes with known roles in genomic stability, as well as dozens of candidate genes with no previously reported role in genomic stability. Thus, the results of our study will provide a foundation for further studies of the molecular mechanisms underlying genomic stability.

Consistent with previous studies reporting increased DNA damage following TDP-43 depletion (Mitra et al., 2019; Konopka et al., 2020), we found elevated DNA damage in iPSCs generated from ALS patients with specific genetic mutations in *TARDBP* and in post-mitotic neurons differentiated from these iPSC lines, suggesting that these defects caused by mutant TDP-43 are not cell-type specific, indicating a molecular consequence of partial loss of TDP-43 function. Although isogenic mutant TDP-43 iPSC lines were not available to us for this study, we observed increased DNA damage selectively in multiple ALS patient iPSC lines but not in multiple control iPSC lines, as well as in *TARDBP* knockdown cells. A potential pitfall of control cell line from healthy individuals is a large heterogeneity due to cell line and genetic differences. Isogenic cell lines from well characterized pre-existing iPSC cell line provides a more accurate interpretation of the data. Most importantly, we observed a strong correlation between TDP-43 nuclear depletion and increased DNA damage in the same neurons from ALS/FTD patient brain tissues. Our findings contribute to the idea that DNA damage is a defining pathological feature of ALS/FTD patients with TDP-43 pathology.

TDP-43 is a key component of the cytoplasmic ubiquitin-positive protein inclusions that are the pathological hallmark of the disease and are found in approximately 97% of ALS patients regardless of genetic etiology (Neumann et al., 2006; Ling et al., 2013). Mutations in TARDBP are uncommon in ALS and have been found in 1-5% of sporadic and familial ALS cases (Mackenzie et al., 2010b; Ravanidis et al., 2018). TDP-43 clearance from the nucleus is almost always linked to the creation of TDP-43-positive cytoplasmic aggregates. We observed a considerable increase in H2AX foci in neurons with decreased nuclear TDP-43 and cytoplasmic aggregation from the ALS patient’s neuron in Figure 4. Our findings suggest TARDBP mutation is not the only cause of higher mutation rate in ALS with TDP-43 pathology, the increased mutation rate is common to ALS/FTD with TDP-43 pathology. Previous studies showed that loss of nuclear TDP-43 function is associated with increased DNA double-strand breaks (Mitra et al., 2019; Giannini et al., 2020; Konopka et al., 2020), however, these studies focused only on the NHEJ DNA repair pathway (Mitra et al., 2019; Konopka et al., 2020). Here we showed that in addition to NHEJ, the HR repair pathway is also misregulated in ALS iPSC-derived neurons. Cells with defects in DNA repair are sensitive to DNA damaging agents and, accordingly, we find that ALS iPSCs and iPSC-derived neurons show a marked reduction in survival following treatment with the DNA damaging agent etoposide or 5-fluorouracil. Our alamar blue cell viability assay shows that cells with TDP43 shRNA grew a little slower than NS shRNA at basal level without DNA damage agents, but not statistical significance (data not shown). Although the HR pathway has been studied mainly in dividing cells, previous reports have shown that HR also occurs in neurons (Cox et al., 2015; Welty et al., 2018; Yu et al., 2018). Our findings expand the role the TDP-43 in maintaining genomic stability beyond the NHEJ pathway and underscore the multi-faceted role of TDP-43 in DNA repair.

Cells frequently sustain DNA lesions that, if not repaired, can have serious effects, including cell death. Since mutations in the DDR and DNA repair genes have been discovered to cause a number of neurodegenerative illnesses, DNA damage and DNA repair deficiencies have long been linked to neurodegeneration. Post-mitotic cells have different DNA repair pathways than mitotic cells. Therefore, DNA repair may be less effective in neuronal cells, increasing the susceptibility of neurons to DNA damage. It has been demonstrated that long-term TDP-43 depletion causes a long-term increase in pATM, persistent DDR activation and eventually cell death (Mitra et al., 2019). Similar to other age-related neurodegenerative diseases, ALS/FTD is linked to an increase in genomic instability. This association is assumed to have a role in the etiology of the disease and the degeneration of motor neurons. Our data are consistent with these findings and support that DNA damage is a primary or secondary factor in the pathogenesis of ALS, not a symptom or consequence.

## Data availability statement

The original contributions presented in the study are included in the article/Supplementary material, further inquiries can be directed to the corresponding author.

## Ethics statement

The studies involving human participants were reviewed and approved by University of California, San Francisco Committee on Human Research. The patients/participants provided their written informed consent to participate in this study.

## Author contributions

MF and MG conceived and designed the experiments. MF designed and conducted the RNAi screen and performed the majority of the experiments. SB and YZ validated some key results. AN and SV performed the histological experiments in FTD-ALS patient brain tissues. SA and F-BG provided iPSC lines and related technical assistance. AW and RB provided key reagents during the early stages of the project. MF, SD, WS, F-BG, and MG analyzed and interpreted the data and wrote the manuscript. All authors reviewed the paper and provided comments.
