# Supplementary material for: Loss of TDP-43 function contributes to genomic instability in amyotrophic lateral sclerosis
Source: Front Neurosci. 2023 Oct 2;17:1251228. doi: 10.3389/fnins.2023.1251228 (PMC10577185; doi:10.3389/fnins.2023.1251228)
Supplement: Supplementary file 1 [file Data_Sheet_1.PDF]

Supplementary Figure Legends

Supplementary Figure 1. Additional experiments related to Figure 1. **(A)** Number of Aprt-/- colonies formed upon plating $1x10^4$, $1x10^5$ or $1x10^6$ 3C4 cells expressing an NS or *Mlh1* shRNA, or parental 3C4 cells, treated in the presence of EMS. The results show that $1x10^4$ *Mlh1* shRNA-expressing 3C4 cells were sufficient to form colonies, whereas $1x10^4$ 3C4 cells expressing a control NS shRNA did not form any colonies. **(B)** *Aprt* mutation rate in mouse 3C4 cells expressing a second shRNA, unrelated to that used in Figure 1B, targeting a selected subset of genes identified in the initial RNAi screen. (*C*) Knockdown efficiencies of two unrelated shRNAs targeting *TARDBP*, *TARDBP-1* and *TARDBP-2*, in A549 cells. Data are represented as mean ± SD; **P*<0.05, ***P*<0.01.

Supplementary Figure 2. Additional experiments related to Figure 2. **(A)** Immunofluorescence showing TUJ1-positive neurons derived from control iPSCs or iPSCs generated from ALS patients harboring either TDP-43(M337V) or TDP-43(Q343R). TUJ1 staining is shown in green, and DAPI staining in blue. **(B)** Comet assay in control neurons expressing a NS shRNA or one of two independent *TARDBP* shRNAs. Representative images are shown on the left, and quantification is provided on the right. Data are represented as mean ± SD; **P*<0.05, ***P*<0.01.

Supplementary Figure 3. Additional experiments related to Figure 3. **(A,B)** Knockdown efficiencies of *TARDBP-1* and *TARDBP-2* shRNAs in HEK293/pPHW1 cells (A) and HCT116/HN5 cells (B). **(C)** Immunofluorescence showing RAD51 staining (green) in A549 cells expressing a NS or *TARDBP-1* shRNA. DAPI staining is shown in blue. Representative images are shown on the left, and quantification is provided on the right. Data are represented as mean ± SD; **P*<0.05, ***P*<0.01.

Supplementary Figure 4. Mutant TDP-43 ALS iPSCs are sensitive to DNA damaging agents. **(A,B)** Survival curves for control, TDP-43(M337V) and TDP-43(Q343R) ALS iPSCs treated with increasing doses of etoposide (*A*) or 5-fluorouracil (*B*). Data are represented as mean ± SD; *$P<0.05$, **$P<0.01$.

Supplementary Table 1. List of mouse and human shRNAs used in this study.

Gene (previous gene name)	First shRNA Clone ID	Second shRNA Clone ID
Aifm3	TRCN0000176611	
Aimp2	TRCN0000099155	TRCN0000099157
Ankrd52	TRCN0000085433	
Atg2b	TRCN0000192436	
Cabs1	TRCN0000178322	
Cdc25c	TRCN0000028954	
Ciao2a (Fam96a)	TRCN0000177038	TRCN0000178357
Crtc2	TRCN0000176130	TRCN0000176098
Cyfip2	TRCN0000097701	TRCN0000097703
Ccn1 (Cyr61)	TRCN0000088186	
Dok2	TRCN0000077430	
E2f5	TRCN0000086469	TRCN0000086468
Fam166c (1700001C02Rik)	TRCN0000191377	
Gm270	TRCN0000024032	
Gpr63	TRCN0000028029	
Gpr171	TRCN0000027558	
Hdac5	TRCN0000039388	TRCN0000039386
Hmx1	TRCN0000070719	TRCN0000070721
Inpp4a	TRCN0000173404	
Kdm1a	TRCN0000071373	TRCN0000071374
Mcf2	TRCN0000042656	TRCN0000042654
Msh2	TRCN0000042496	
Msh3	TRCN0000174683	
Ncoa2	TRCN0000096169	TRCN0000096170
Nme1	TRCN0000024733	
Nprl2	TRCN0000042422	
Nrip3	TRCN0000198805	
Nsrp1	TRCN0000123529	
Oit3	TRCN0000097650	
Olfm2	TRCN0000127405	TRCN0000127406
Or2g1 (Olfr123)	TRCN0000187154	
Or4f61 (Olfr1314)	TRCN0000030160	
Or51r1 (Olfr550)	TRCN0000189098	
Or6c3 (Olfr788)	TRCN0000185809	
Or7c19 (Olfr371)	TRCN0000185090	
Or8d23 (Olfr930)	TRCN0000187675	
Or8k3b (Olfr1087)	TRCN0000185759	
Or9i2 (Olfr1501)	TRCN0000186608	
Pik3r2	TRCN0000025088	TRCN0000025086
Poteg	TRCN0000103810	
Prkd3	TRCN0000024185	
Prrxl1	TRCN0000070425	
Prss41	TRCN0000033009	TRCN0000033010
Prss50	TRCN0000031381	
Rspry1	TRCN0000040682	
Rxrb	TRCN0000027090	

Sigmar1	TRCN0000194052	
Slc51b	TRCN0000079747	
Sptbn5	TRCN0000091407	
Strap	TRCN0000088837	TRCN0000088835
Tacc1	TRCN0000126701	TRCN0000126702
Tal1	TRCN0000042573	TRCN0000042574
Tardbp (mouse)	TRCN0000173538	TRCN0000174930
TARDBP (human)	TRCN0000016038	TRCN0000016041
Tcta	TRCN0000126625	TRCN0000126627
Tescl	TRCN0000182116	
Tmed10	TRCN0000112381	
Tmem126b	TRCN0000192549	
Tpd52l1	TRCN0000088395	TRCN0000088394
Trhr2	TRCN0000028178	
Tssk4	TRCN0000024239	
Usp28	TRCN0000030867	
Vrk2	TRCN0000023760	TRCN0000023759
Zfhx4	TRCN0000075531	TRCN0000075529
Zfp37	TRCN0000084382	TRCN0000084381
Zfp113	TRCN0000085981	
Zfp704	TRCN0000111976	TRCN0000111975
Zfp935	TRCN0000174822	
Zmynd8	TRCN0000088513	
BC053393	TRCN0000100100	
4931428L18Rik	TRCN0000182788	

Supplementary Table 2. List of normal and patient samples and information.

Subject	Gene status	Sex	Age at death (years)	PMI (hours)	Clinical diagnosis	Primary neuropathological diagnosis	ADNC level	Region(s) studied
C1	C9orf72	M	68	7.5	bvFTD-MND	FTLD-TDP-B, MND	Not ADNC	PreCG
C2	C9orf72	M	58	7.5	bvFTD-MND	FTLD-TDP-U, MND	Low	FI
C3	C9orf72	M	57	11.5	bvFTD	FTLD-TDP-B, MND	Low	PreCG, FI
S1		M	66	12.1	FTD-MND	FTLD-TDP-B, MND	Low	PreCG, IFG
S2		F	65	8.5	bvFTD	FTLD-TDP-B; MND	Low	FI
S3		M	72	6.7	bvFTD-MND	FTLD-TDP-B	Not ADNC	PreCG, FI
S4		M	56	12.9	nfvPPA-ALS	FTLD-TDP-B, MND	Not ADNC	IFG
NC		M	76	8.2	Control	None	Low	IFG

ADNC: Alzheimer's disease neuropathologic change; ALS: amyotrophic lateral sclerosis; bvFTD: behavioral variant frontotemporal dementia; C: C9orf72-associated disease; F: female; FI: frontoinsular cortex; FTLD-TDP: frontotemporal lobar degeneration with TDP-43 inclusions; FTD: frontotemporal dementia; IFG: inferior frontal gyrus; M: male; MND: motor neuron disease; NC, neurologically unaffected control; nfvPPA: nonfluent variant primary progressive aphasia; PMI: post-mortem interval; PreCG: precentral gyrus; S: sporadic disease.

Supplementary Table 3. List of genes identified in the primary RNAi screen.

**Biological process**	**Mouse gene symbol**	**Human gene symbol**	**Gene name**
Apoptosis and autophagy	*Aifm3*	*AIFM3*	Apoptosis inducing factor mitochondria associated 3
	Aimp2	*AIMP2*	Aminoacyl tRNA synthetase complex interacting multifunctional protein 2
	Atg2b	*ATG2B*	Autophagy related 2B
Cell cycle regulation	*Cdc25c*	*CDC25C*	Cell division cycle 25C
	Tacc1	*TACC1*	Transforming acidic coiled-coil-containing protein 1
Cell differentiation	*Olfm2*	*OLFM2*	Olfactomedin 2
Chromosome segregation	*Ciao2a*	*CIAO2A*	Cytosolic iron-sulfur assembly component 2A
DNA damage/repair	*Msh2*	*MSH2*	mutS homolog 2
	Msh3	*MSH3*	mutS homolog 3
	Usp28	*USP28*	Ubiquitin specific peptidase 28
Nucleic acid metabolism	*Nme1*	*NME1*	NME/NM23 nucleoside diphosphate kinase 1
	Nsrp1	*NSRP1*	Nuclear speckle splicing regulatory protein 1
	Tardbp	*TARDBP*	TAR DNA binding protein
Protein metabolism	*Nrip3*	*NRIP3*	Nuclear receptor interacting protein 3
	Prss41	*PRSS41*	Serine protease 41
	Prss50	*PRSS50*	Serine protease 50
Signal transduction	*Ankrd52*	*ANKRD52*	Ankyrin repeat domain 52
	Cabs1	*CABS1*	calcium binding protein, spermatid associated 1
	Cyfip2	*CYFIP2*	cytoplasmic FMR1 interacting protein 2
	Ccn1	*CCN1*	cellular communication network factor 1
	Dok2	*DOK2*	Docking protein 2
	Gpr63	*GPR63*	G protein-coupled receptor 63
	Gpr171	*GPR171*	G protein-coupled receptor 171
	Inpp4a	*INPP4A*	Inositol polyphosphate-4-phosphatase type IA
	Mcf2	*MCF2*	MCF.2 cell line derived transforming sequence
	Nprl2	*NPRL2*	NPR2 like, GATOR1 complex subunit
	Oit3	*OIT3*	Oncoprotein induced transcript 3
	Or1o11	—	Olfactory receptor family 1 subfamily O member 11
	Or2g1	—	Olfactory receptor family 2 subfamily G member 1
	Or4f61	—	Olfactory receptor family 4 subfamily F member 61
	Or6c3	—	Olfactory receptor family 6 subfamily C member 3
	Or7c19	—	Olfactory receptor family 7 subfamily C member 19
	Or8d23	—	Olfactory receptor family 8 subfamily D member 23
	Or9i2	—	Olfactory receptor family 9 subfamily I member 2

	Or51r1	—	Olfactory receptor family 51 subfamily R member 1
	Pik3r2	*PIK3R2*	Phosphoinositide-3-kinase regulatory subunit 2
	Prkd3	*PRKD3*	Protein kinase D3
	Sigmar1	*SIGMAR1*	Sigma non-opioid intracellular receptor 1
	Strap	*STRAP*	Serine/threonine kinase receptor associated protein
	Tpd52l1	*TPD52L1*	Tumor protein D52-like 1<br>TDP52 like 1 (human)
	Trhr2	—	Thyrotropin releasing hormone receptor 2
	Tssk4	*TSSK4*	Testis specific serine kinase 4
	Vrk2	*VRK2*	Vaccinia related kinase 2<br>VRK serine/threonine kinase 2 (human)
Transcription regulation	*Crtc2*	*CRTC2*	CREB regulated transcription coactivator 2
	E2f5	*E2F5*	E2F transcription factor 5
	Hdac5	*HDAC5*	Histone deactylase 5
	Hmx1	*HMX1*	H6 family homeobox 1
	Kdm1a	*KDM1A*	Lysine demethylase 1A
	Ncoa2	*NCOA2*	Nuclear receptor coactivator 2
	Prrxl1	*DRGX*	Paired related homeobox protein-like 1<br>Dorsal root ganglia homebox (human)
	Rspry1	*RSPRY1*	RING finger and SPRY domain-containing protein 1
	Rxrb	*RXRB*	Retinoid X receptor beta
	Tal1	*TAL1*	TAL bHLH transcription factor 1, erythroid differentiation factor
	Zfhx4	*ZFHX4*	Zinc finger homeobox 4
	Zfp37	*ZFP37*	ZFP37 zinc finger protein
	Zfp113	*ZNF3*	Zinc finger protein 113<br>Zinc finger protein 3 (human)
	Zfp704	*ZNF704*	Zinc finger protein 704
	Zfp935	—	Zinc finger protein 935
	Zmynd8	*ZMYND8*	Zinc finger MYND-type containing 8
Transport/ Trafficking	*Slc51b*	*SLC51B*	Solute carrier family 51 beta subunit
	Sptbn5	*SPTBN5*	Spectrin beta, non-erythrocytic 5
	Tmed10	*TMED10*	Transmembrane p24 trafficking protein 10
	Tmem126b	*TMEM126B*	Transmembrane protein 126B
Unknown	*Fam166c*	*FAM166C*	family with sequence similarity 166 member C
	Gm270	—	Predicted gene 270
	Poteg	*POTEG*	POTE ankyrin domain family member G
	Tcta	*TCTA*	T-cell leukemia translocation-altered
	Tescl	—	Tescalcin-like
	BC053393	—	cDNA sequence BC053393
	4931428L18Rik	—	RIKEN cDNA 4931428L18
