# Supplementary figures and images for: Loss of TDP-43 function contributes to genomic instability in amyotrophic lateral sclerosis
Source: Front Neurosci. 2023 Oct 2;17:1251228. doi: 10.3389/fnins.2023.1251228 (PMC10577185; doi:10.3389/fnins.2023.1251228)

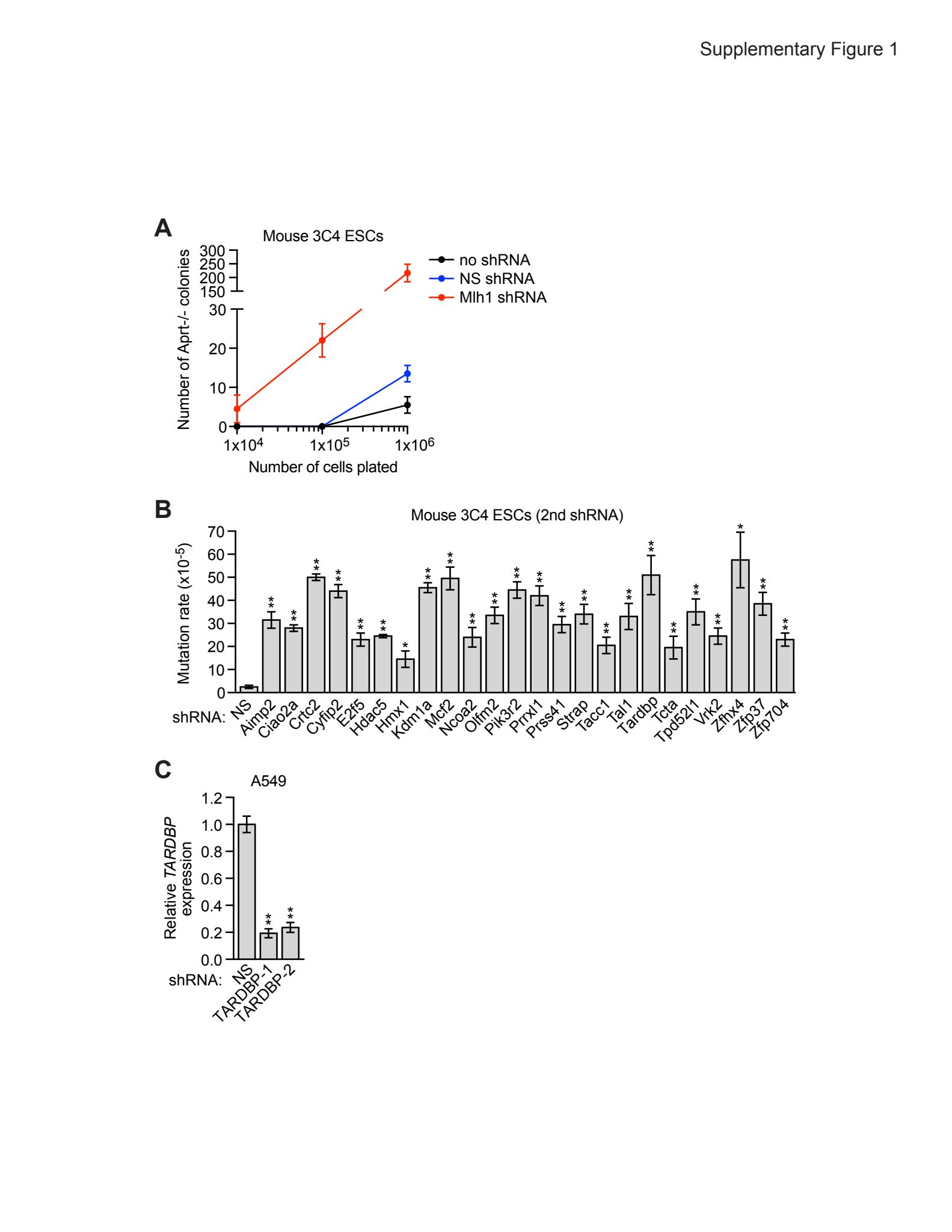

Supplement: Supplementary file 2 [file Image_1.JPEG]

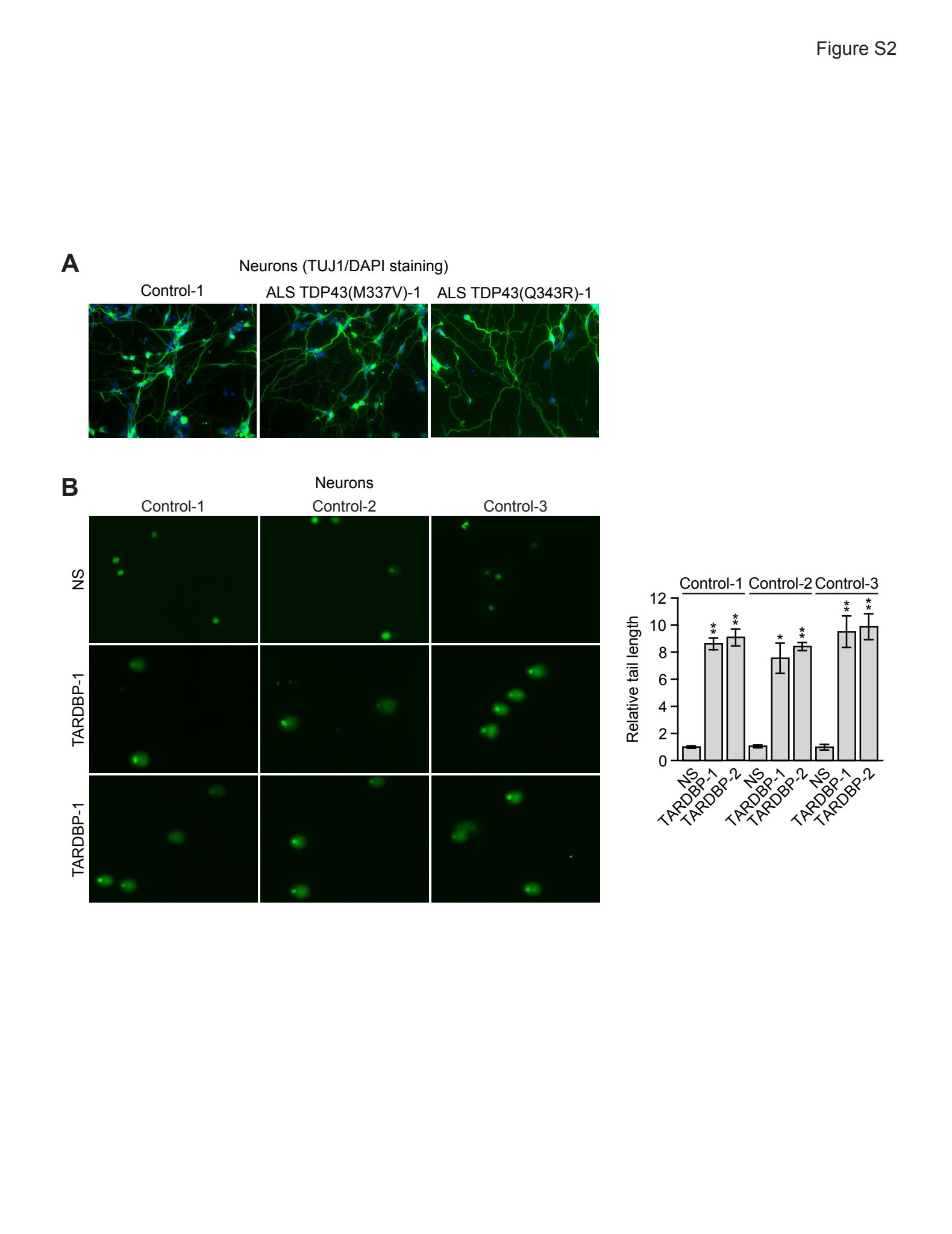

Supplement: Supplementary file 3 [file Image_2.JPEG]

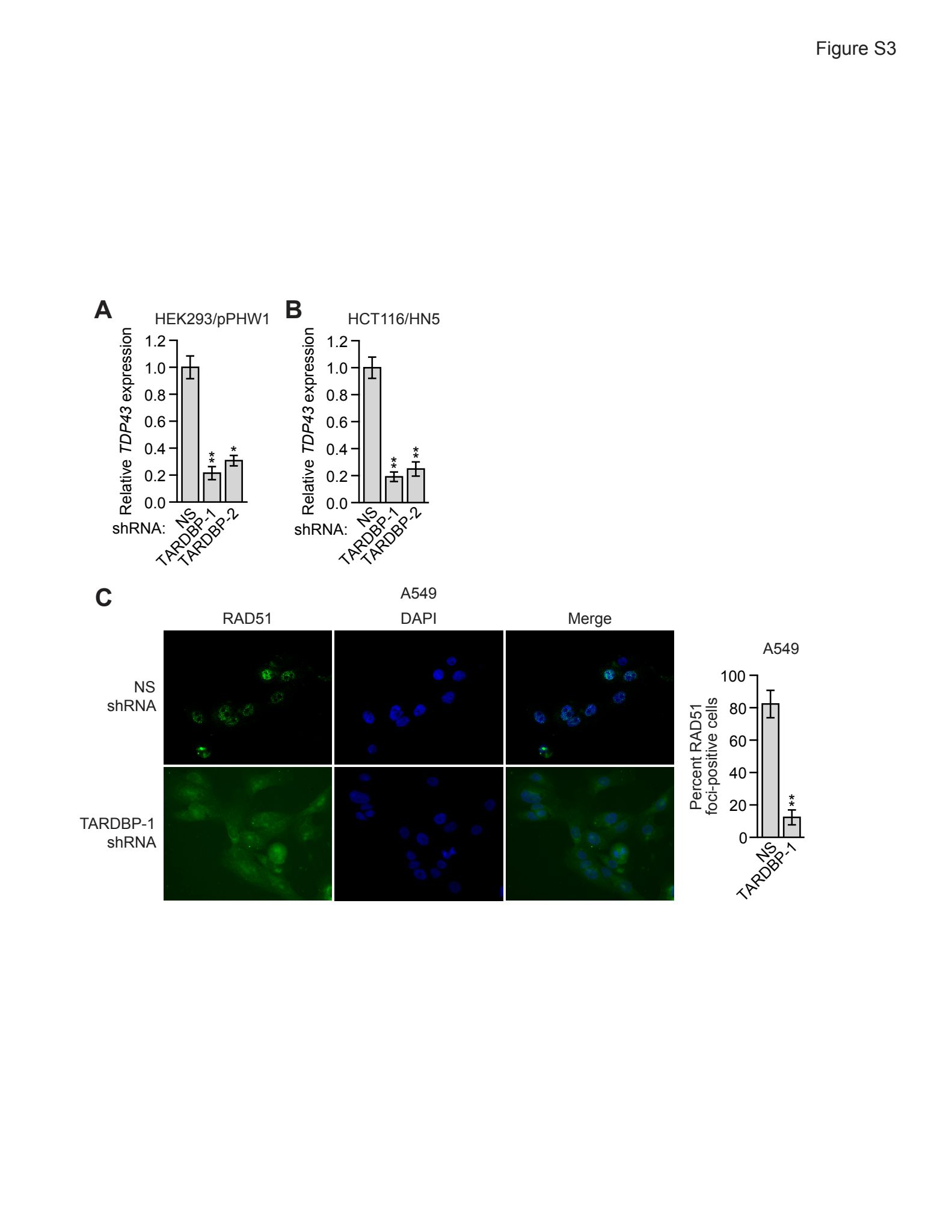

Supplement: Supplementary file 4 [file Image_3.JPEG]

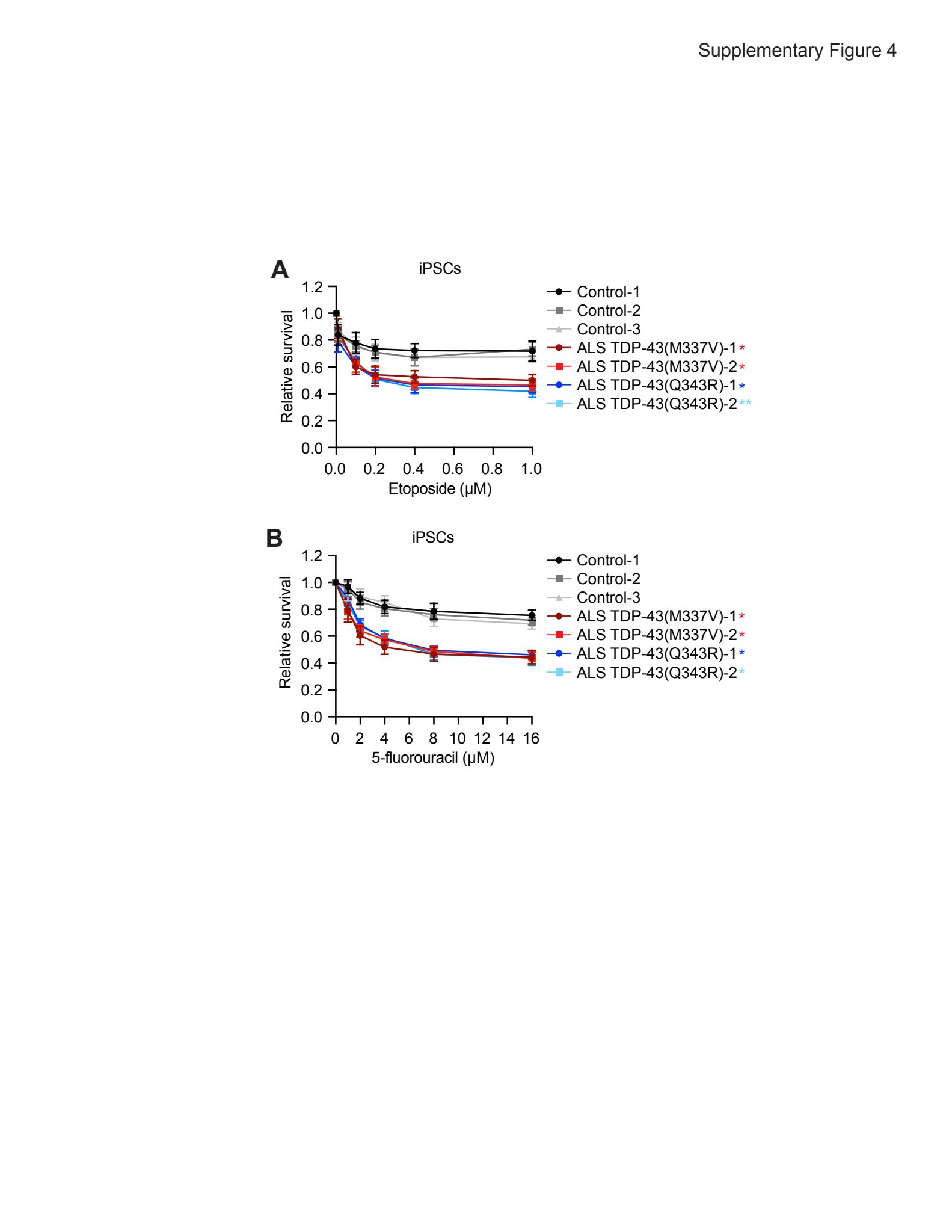

Supplement: Supplementary file 5 [file Image_4.JPEG]
